# Renal Toxicogenomic Response to Chronic Uranyl Nitrate Insult in Mice

**DOI:** 10.1289/txg.7296

**Published:** 2004-10-15

**Authors:** Magali Taulan, François Paquet, Christophe Maubert, Olivia Delissen, Jacques Demaille, Marie-Catherine Romey

**Affiliations:** ^1^Institut de Radioprotection et de Sûreté Nucléaire, Laboratoire de Radiotoxicologie Expérimentale, Pierrelatte, France; ^2^Institut de Génétique Humaine, Laboratoire de Génétique Moléculaire et Chromosomique, Montpellier, France

**Keywords:** drinking water, gene expression profiles, long-term exposure, mice, SAGE, toxicogenomics, uranyl nitrate

## Abstract

Although the nephrotoxicity of uranium has been established through numerous animal studies, relatively little is known about the effects of long-term environmental uranium exposure. Using a combination of conventional biochemical studies and serial analysis of gene expression (SAGE), we examined the renal responses to uranyl nitrate (UN) chronic exposure. Renal uranium levels were significantly increased 4 months after ingestion of uranium in drinking water. Creatinine levels in serum were slightly but significantly increased compared with those in controls. Although no further significant differences in other parameters were noted, substantial molecular changes were observed in toxicogenomic profiles. UN induced dramatic alterations in expression levels of more than 200 genes, mainly up-regulated, including oxidative-response–related genes, genes encoding for cellular metabolism, ribosomal proteins, signal transduction, and solute transporters. Seven differentially expressed transcripts were confirmed by real-time quantitative polymerase chain reaction. In addition, significantly increased peroxide levels support the implication of oxidative stress in UN toxicant response. This report highlights the potential of SAGE for the discovery of novel toxicant-induced gene expression alterations. Here, we present, for the first time, a comprehensive view of renal molecular events after uranium long-term exposure.

Uranium, the heaviest of the naturally occurring elements, is widely present in the environment as a result of leaching from natural deposits, release in mill tailings, emissions from the nuclear industry, the combustion of coal and other fuels, and the use of phosphate fertilizers and weapons that contain uranium. Thus, uranium is found in various chemical forms and different levels in all soils, rocks, sea, and bedrock (Bosshard et al. 1992; Kurttio et al. 2002; Moss et al. 1983). It is also found in both food and drinking water. The wide range of levels of uranium in drinking water, together with the observation of consistently higher levels in certain community water supplies, has raised concerns regarding the potential hazard of such sources of uranium to human health.

Many isolated studies conducted on the mechanisms for the toxic effects of uranium at moderate to high acute doses on experimental animals have shown that the major health effect of uranium is chemical kidney toxicity rather than a radiation hazard (Lin et al. 1993; Miller et al. 1998, 2002). In addition only a few studies have attempted to characterize the effects of chronic exposure to uranium through drinking water (Gilman et al. 1998a, 1998b, 1998c; Kurttio et al. 2002; McDonald-Taylor et al. 1997; Zamora et al. 1998). Although chronic uranium exposure in humans has been clearly associated with increasing urinary glucose, alkaline phosphatase, and β_2_-microglobulin supporting proximal tubule alterations, the urinary albumin levels, which are indicators of glomerular function, are conflicting (Kurttio et al. 2002; Zamora et al. 1998). Although both functional and histologic damage to the proximal tubules resulting from acute uranium exposure has been clearly demonstrated (Schramm et al. 2002; Sun et al. 2002), little is known about the effect of long-term environmental uranium exposure in both humans and animals (Gilman et al. 1998a, 1998b, 1998c; Kultima et al. 2002; Mao et al. 1995; McDonald-Taylor et al. 1997; Zamora et al. 1998).

Toxicogenomics is presently used to evaluate risk assessment of environmental toxicants through the identification of gene expression networks, as well as to evaluate toxicant-induced gene expression as a biomarker to assess human exposure. Several researchers are currently combining the identification of gene expression patterns representative of adverse outcomes with traditional biochemical parameter measures to categorize and classify toxic responses through direct comparison in exposed and control samples. The use of oligonucleotide-based or cDNA microarrays for understanding the biochemical processes associated with environmental chemical exposures has proven successful in recent experiments on human health risk assessment for several toxicants (Andrew et al. 2003; Bartosiewicz et al. 2001).

Because the risk assessment and establishment of exposure limits for uranium in drinking water are of considerable importance in various areas, including Finland, we used for the first time the SAGE (serial analysis of gene expression) approach to identify gene expression profiles associated with this hazard exposure. Because toxicogenomics provides increased confidence in extrapolation of hazards observed in animals studies to likely hazards in humans, we examined renal molecular effects of chronic exposure to uranium in mice.

## Materials and Methods

### Animals

The C57BL/6J mouse was chosen because of the current state of knowledge about this transcriptome and numerous databases such as Mouse SAGE Site (http://mouse.biomed.cas.cz/sage/). This animal model should help improve the overall quality of SAGE gene expression data. Experiments were performed with 16 male C57BL/6J mice, weighing 25–30 g (Harlan, Gannat, France) at the beginning of the study. The mice were randomly divided into three groups: one control group (group 0, six animals) and two uranyl nitrate (UN)-treated mice (groups 1 and 2, six and four animals, respectively). Exposed groups 1 and 2 received UN mineral water at concentrations of 80 or 160 mg UN/L of water, respectively, approximately 3- or 6-fold higher than levels found in bedrock of southern Finland (Juntunen 1991). Uranium in water, given to control mice, was determined to be < 0.002 mg/L uranium. Body weights were measured weekly. Food intake and fluid consumption data were recorded. After 4 months of treatment all animals were euthanized by exsanguination using cardiac puncture. Urine and blood were collected for each group. The kidneys were either embedded in Epon for morphologic examination or snap-frozen in liquid nitrogen and then stored at −70°C until further study.

### Assessment of Renal Function Parameters

Uranium contents were determined in samples of kidney using a kinetic phosphorescence analyzer (KPA; Ejnik et al. 2000). Serum creatinine and urea levels and urinary concentrations of glucose and γ-glutamyl transpeptidase (γ-GT) were measured by routine methods.

### RNA Isolation

Total RNAs, extracted from renal tissue using the RNA isolation mini kit (Qiagen, Courtaboeuf, France), were pooled for SAGE or used individually for real-time reverse transcriptase polymerase chain reaction (RT-PCR) analyses. The amount of total RNA was determined using a fluorescent nucleic acid stain (RiboGreen RNA Quantitation Kit; Molecular Probes, Montluçon, France). The quality of the RNA was evaluated by measuring the 260:280-nm ratios and confirmed by visualization of intact 18S and 28S RNA bands after agarose gel electrophoresis.

### Analysis of Gene Expression

#### Production of kidney library.

Kidney libraries were generated from 50 μg of total RNA using the I SAGE kit (Invitrogen Corp., Cergy Pontoise, France) following the manufacturer’s instructions (Invitrogen Corporation 2004), adapted from initial description (SAGE 2004; Velculescu et al. 1995). Because of budgetary restrictions, SAGE was performed for only control [UN(−)] and 80 mg UN/L–treated mice [UN(+)], that is, groups 0 and 1, respectively.

#### Tag quantification.

Concatemer sequences were analyzed by using SAGE software (version 4.0; Invitrogen), which automatically detects and counts tags from sequence files. SAGE software excludes replicate ditags from the tag sequence catalog because the probability of any two tags being coupled in the same ditag is small, even for abundant transcripts. For tag identification, the tag list of each library was matched against a mouse tag database extracted by SAGE software from GenBank (http://www.ncbi.nlm.nih.gov/). Usually, SAGE tag sequences matched more than one transcript. The average *p*-value computed by the SAGE software, based on a Monte Carlo analysis (Zhang et al. 1997), serves as ranking parameter to produce a list of differentially expressed genes. SAGE data for the libraries described here are available at Gene Expression Omnibus (www.ncbi.nlm.nih.gov/geo); accession nos. GSM24256 and GSM24257).

### Real-Time RT-PCR

Total RNAs (1 μg) from UN(−) and UN(+) renal tissue (extracted as described above) were used to generate cDNA using Moloney Murine Leukemia Virus Reverse Transcriptase (Invitrogen) according the manufacturer’s conditions. Primers and probes specifically designed for selected cDNA using Primer Express software, version 2.0 (PE; Applied Biosystems, Courtaboeuf, France), are listed in Table 1. The ABI PRISM 7000 Sequence Detection System was used for detected real-time RT-PCR products with the SYBR Green I assay, according to recommendations of the manufacturer (PE; Applied Biosystems). For two cases in which we encountered difficulties with the SYBR Green I assay, we used TaqMan probe assays (Applied Biosystems) (Table 1). Each PCR reaction was optimized to ensure that a single band of the appropriate size was amplified and that no bands corresponding to genomic DNA amplification or primer–dimer pairs were present. The PCR cycling conditions were performed for all samples as follows: 50°C, 2 min for AmpErase UNG (Applied Biosystems) incubation; 95°C, 10 min for AmpliTaq Gold (Applied Biosystems) activation; and 40 cycles for the melting (95°C, 15 sec) and annealing/extension (60°C for 1 min) steps. PCR reactions for each template were done in triplicate in 96-well plates. The comparative threshold cycle (*C_t_*) method using Primer Express software, version 2.0, was used to determine relative quantitation of gene expression for each gene compared with the hypoxanthine guanine phosphoribosyl transferase control (listed in Table 1).

### Hydrogen Peroxide Assay

To determine the impact of UN on the oxidative balance status, hydrogen peroxide levels were determined using a PeroxiDetect kit (Sigma, Lyon, France). Briefly, kidney samples from different groups (0, 1, and 2) were homogenized in the indicated phosphate buffer on ice, then centrifuged at 15,000×*g* for 15 min at 4°C. Supernatant samples (100 μL) were incubated for 30 min with 1 mL of aqueous peroxide color reagent (aqueous solution containing 100 mM sorbitol and 125 μM xylenol orange) and 10 μL of ferrous ammonium sulfate reagent (25 mM ferrous ammonium sulfate in 2.5 M sulfuric acid), and the hydrogen peroxide level was measured by the absorbance at 560 nm.

## Results

### General Observations

To examine the general parameters, we performed gross end-point analysis such as body and organ weight changes and histologic observations, as well as the dosage of uranium content in renal tissue and biochemical markers. No significant dose-related effects were observed on body weight gain, food intake, or water consumption. Because the concentrations of UN in the drinking water remained constant throughout the study, it is natural to assume that the measurement of UN per kilogram body weight decreased with age.

Gross pathologic examination was performed in all animals, and the histopathologic analysis did not identify any significant differences between control and exposed groups. We observed a significant dose-dependent increase in renal uranium tissue levels in groups 1 and 2 compared with control mice, using KPA. Compared with controls, there were no significant differences in kidney weights in any dose group (Table 2). Serum creatinine levels appeared to increase in dose-independent manner with UN treatment, and groups 1 and 2 showed creatinine levels significantly higher than those of controls.

### Genes Responding to Toxic UN Exposure

We investigated the transcriptomic response that underlies the induction of the metal-elicited molecular modification in C57/Bl6J mice. SAGE was used to determine the global gene expression profile in UN toxicity. This approach allows an analysis of gene expression by the sequencing of approximately 21,000 transcripts from kidney libraries of the groups 0 and 1, which represent 5,252 and 4,069 unique tags, respectively.

We validated the quality of both libraries by comparing both with previous data on the kidney (Chabardes-Garonne et al. 2003; El-Meanawy et al. 2000; Virlon et al. 1999). For example, known markers for proximal tubules [kidney androgen-regulated protein (*kap*)] and thick ascending limbs [uromodulin (*Umod*)] were evidenced in both libraries. As expected, a large fraction of the most abundant tags matched with widely expressed mitochondrial genes or ribosomal proteins such as ribosomal proteins P1 and S26. Because the kidney mass consists predominantly of proximal tubules, a significant fraction of tags are mapped to genes known to be expressed in proximal tubular epithelial cells. Particularly, the most abundant transcripts in normal kidney were *kap* and glutathione peroxidase 3 (*GPx3*), in agreement with previous data (El-Meanawy et al. 2000).

Tags that are significantly up- or down-regulated in the UN RNA library are listed in Table 3 with their frequency and their relevant accession number. We considered only the transcripts with a significant expression change (*p* < 0.05). Considering the large number of sequenced tags, the number of genes expressed in kidney was evaluated by excluding tags matching mitochondrial sequences, tags with multiple matches, and nonreliable matches. Tags were arbitrarily separated in categories according to gene function. As illustrated in Table 3, most of these changes involved up-regulation. SAGE analysis revealed the expression changes of genes related to lipid metabolism [crystalline, zeta (*Cryz*); phosphatidic acid phosphatase type 2c (*PPap2c*)], carbohydrate metabolism [phosphoglycerate kinase 1 (*Pgk1*); sorbitol dehydrogenase 1 (*Sdh1*)], and amino acid metabolism (glutamate dehydrogenase (*Glud*); ornithine decarboxylase, structural (*Odc*). The UN-induced transcripts consisted mainly of genes encoding proteins associated with protein biosynthesis [ribosomal protein S25 (*Rps25*); S26 (*Rps26*); large, P1 (*Rplp1*); L19 (*Rpl19*)], protein folding [heat-shock 10 kDa protein 1 (chaperonin 1) (*Hspe1*)], and proteolysis [kallikrein 5 (*Klk5*), protein C (Proc)]. Many genes involved in signaling were up-regulated, such as hormonal receptors [growth hormone receptor (*Ghr*), cholecystokinin A receptor (*Cckar*)]. Chronic exposure to UN also increased the expression of a number of genes related to oxidative process and detoxification. Among these is cytochrome P450 (*Cyp4b1*), which catalyzes the oxidation of a wide variety of substrates, including endogenous lipids and xenobiotics (Heng et al. 1997). Other relevant enzymes under- or overexpressed include thioredoxin, mitochondrial (*Txn2*); superoxide dismutase 1, soluble (*Sod1*); and thioether *S*-methyltransferase (*Temt*). We also mainly observed up-regulation of genes related with ion transporters including solute carrier family 34 (sodium phosphate), member 1 (*Slc34a1, NaPi-II*); and with electron transporters such as ATPase inhibitor, and cytochrome *c* oxidase, subunit IVa (*Cox4a*); subunit VIIIa (*Cox8a*); and subunit XVII assembly protein homolog (*Cox 17* ). Finally, expression levels of several genes, in the category related to stress/apoptosis [Bcl2 associated athanogene 1 (*Bag1*); nerve growth factor receptor (TNFRSF16) associated protein 1 (*Ngfrap1*)]; immunity (Ia-associated invariant chain (*Ii*)]; and translationally regulated transcripts (21 kDa) (*Trt, Tpt1, Tctp, Umod*) were changed.

### Real-Time Quantitative PCR Analyses

**Figure 1:**
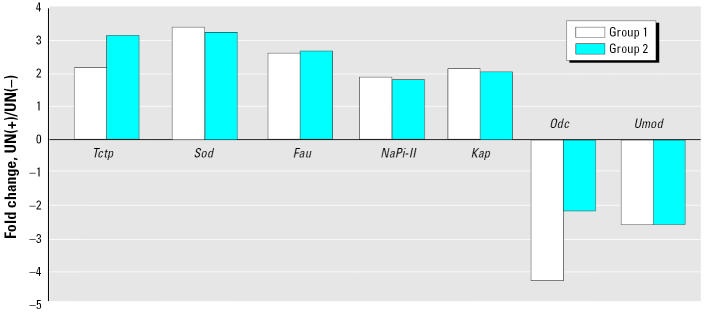
Confirmation of SAGE data by real-time RT-PCR analysis. The variation of the amplification of the expression in groups 1 and 2 [UN(−)/UN(+)] is plotted. PCR analyses were performed on cDNA from UN(−) or UN(+) tissues.

To validate our SAGE data, we conducted real-time quantitative PCR analyses to verify the differential expression of seven selected genes (Figure 1). *kap* was chosen because of its high abundance level in the normal and contaminated kidney. Solute carrier family 34 [sodium phosphate, NaPi] member 1 (*slc34a1*, *NaPi-II*)], *Sod1*, Finkel-Biskis-Reilly murine sarcoma virus ubiquitously expressed (*Fau*), and translationally regulated transcript (*Trt* or *Tctp*) were chosen because they were increased in our data. *Umod* and ornithine decarboxylase structural (*Odc*) were chosen because their expression levels were decreased in the present study as well as in ischemic acute renal failure (ARF) or UN-induced chronic renal failure, respectively (Fleck et al. 2003). Using real-time PCR analyses, *Kap*, *NaPi-II*, *Sod*, *Fau*, and *Tctp* were confirmed to be significantly increased whereas *Odc* and *Umod* were decreased in chronic exposure to UN. In summary PCR analysis confirmed the accuracy of the differences in expression levels observed in our SAGE analysis for group 1. Moreover, using real-time PCR for group 2, we observed that the expression of the selected transcripts were altered in the same direction compared with group 1, that is, increased or decreased. We noted dose-dependent increases in *Tctp* mRNA level at the highest concentration, and the observed decrease of *Odc* mRNA levels was more moderate for group 2.

### Peroxide Level Measurement

**Figure 2:**
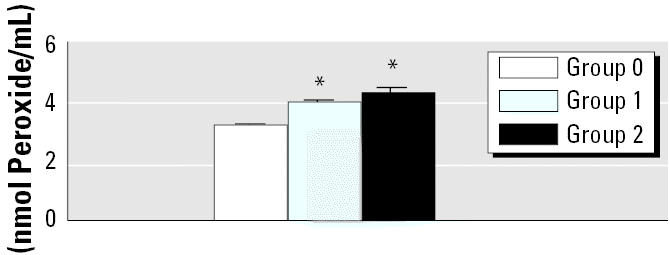
Measurement of hydrogen peroxide already formed in kidney tissue. An increase in H_2_O_2_ level was induced by UN in a dose-dependent manner. Data shown represent means ± SE of three independent experiments (*n* = 4).
*p < 0.05 versus control.

To evaluate whether the variations in both *Sod* and *Gpx* transcripts may reflect a potential oxidative stress, we examined the production of H_2_O_2_. The concentration of H_2_O_2_ in the kidney was found to be significantly higher in groups 1 and 2 compared with the control group (4.06 ± 0.06 and 4.39 ± 0.11 vs. 3.3 ± 0.02 nmol peroxide/mL) (Figure 2). Long-term UN exposure clearly caused the production of H_2_O_2_ levels in UN groups 1 and 2, in dose-dependent fashion.

## Discussion

Human exposures to metals such as uranium in both occupational and environmental settings are common occurrences. Uranium exposures are a growing concern in our society. Classically, toxicologists assess potential chronic adverse health outcomes resulting from chemical exposures by using gross end points such as body or organ weight changes and histopathologic observations. However, analysis of histologic or biochemical markers often does not provide information about the mechanisms involved in toxicant response. The study of molecular mechanisms of toxicant action might provide information crucial to the understanding of their potential adverse effects on human health. Recent technologies such as SAGE facilitate studies that add insight into the cellular response to chemical exposure. In environmental monitoring, SAGE could not only provide a method for quickly categorizing chemicals and assigning a mode of toxic action but also allow more sensitive end points to address specifically gene expression pattern.

Results reported here identify > 200 genes from approximately 21,000 tags sequenced, for which the expression in kidney changed significantly after UN long-term exposure. Most of these tags represent distinct transcripts; however, some tags, especially those detected only once, may result from PCR or sequencing errors (Velculescu et al. 1997; Zhang et al. 1997). Using classical end-point examination, including histologic appearance of the kidney and clinical and biochemical parameters, we observed that the UN doses used in this study produced only a slight alteration in serum creatinine levels and a significant but nonlinear increase of intrarenal uranium content. The dose-independent induction of the serum creatinine may be attributable, as already reported (Amin et al. 2004), to the fact that this parameter, like serum urea, traditionally used as indices of changes in glomerular filtration rate, is a relatively insensitive marker of glomerular injury. Taken together, these data suggest that the glomerular filtration rate remains relatively normal in mice after UN chronic exposure. Because the degree of renal injury appeared to be minimal in the strain of mouse used in the present study, further work will be needed to correlate the renal toxicity with the chronic uranium treatment, in dose- and time-dependent manner.

At the molecular level we observed that UN induced changes in expression profiles for oxidative response–related genes and genes encoding for ribosomal proteins, cellular metabolism, signal transduction, and solute transporters. Altered expression of these genes likely reflects an altered protein product (not determined in the present study).

### Oxidative Stress Response

Reactive oxygen species (ROS) are produced by the metabolism of O_2_ in all aerobic cells and are essential for normal cellular signaling functions. However, oxidative stress can occur as a result of either increased ROS generation or depressed antioxidant system, or both. Of them, SOD, catalase, and GPx constitute the main components of the antioxidant defense system. These antioxidants protect the cell against cytotoxic ROS such as superoxide anions, hydrogen peroxide, and hydroxyl radicals. The measurement of peroxides in biologic systems is one of the factors allowing the determination of the degree of certain free radicals present in specific tissues. Recently, Jung et al. (2003) suggested that H_2_O_2_ produced by arsenite might activate growth factor receptor by increasing its tyrosine phosphorylation. These data indicated that H_2_O_2_ might be a pivotal mediator of the tumor-promoting activity of arsenite (Jung 2003). In the present study we observed that UN induces dose-dependent production of H_2_O_2_. We also observed an increase in Cu,Zn-SOD mRNA levels in the kidney. SOD is an enzyme responsible for dismutation of highly reactive superoxide radicals to H_2_O_2_. Moreover, GPx, which scavenges H_2_O_2_ and lipid peroxides, had its gene expression level increased, potentially induced by the high concentrations of H_2_O_2_. Induction of oxidative balance perturbation has been previously described in UN-induced ARF (Schramm et al. 2002). In addition, it has also been reported that some toxicants such as cadmium and arsenic are able to induce an increase in H_2_O_2_ levels after acute exposure (Ercal et al. 2001). Taken together, these data suggest that UN induces oxidative stress. Exploring this point seems of interest in evaluating the risks of UN long-term exposures.

### Involvement of Genes Encoding Ion Transporters

The proximal tubule (especially the S3 segment) and the outer medullary thick ascending limb suffer the most severe injury after toxic and ischemic insult (Kwon et al. 2000; Sun et al. 2000). Although basolateral transport of sodium among the entire nephron and collecting ducts occurs via the active Na-K-ATPase pump, the active absorption is mediated by the Na^+^-dependent inorganic phosphate co-transporters (NaPi-II). In contrast to a previous study (Park et al. 1997) showing that chronic exposure to cadmium impairs the Pi transport capacity, probably by reducing the effective number of *NaPi* co-transporter units, we found that UN long-term exposure induces an increase of NaPi-II mRNA levels. As already suggested (Levi et al. 1994; Loghman-Adham 1997), this increase in NaPi-II is probably the result of an increase in *V*_max_ by a transporter-shuttling mechanism, which is sensitive to disruptors of microtubule integrity. In addition, as previously reported (Moz et al. 1999) in hypophosphatemia studies, our observations suggest that UN chronic exposure could enhance the renal translational machinery. Further experiments, for example, examining the *in vivo* rates of NaPi-II synthesis, should allow clarification of whether UN-like hypophosphatemia affects NaPi-II translation. Moreover, *Na-K-ATPase* expression levels are down-regulated after UN long-term ingestion. This observation is consistent with previous work, after ischemic injury, that also shows a decreased *Na-K-ATPase* mRNA transcription (Kwon et al. 2000). The potential significance of this observation is that urine volume might be increased because of decreased Na^+^ reabsorption. Unfortunately, urine volumes were not recorded throughout the experiments, and the monitoring of the water consumption did not reveal any change in differently treated groups compared with controls. Thus, the role of these proteins in response to UN exposure remains unclear and warrants additional investigation.

### Involvement of Protein Biosynthesis–Related Genes

Interestingly, many ribosomal subunits and other factors involved in protein synthesis (elongation factor) were induced upon UN treatment. Ribosomal proteins are major component of ribosomes that catalyze protein biosynthesis in the cytoplasm of cells. Under normal growth conditions, ribosomal proteins are synthesized stoichiometrically, in relation with ribosomal RNA, to produce an equimolar supply of ribosomal components. However, regulation of the transcriptional activity of the genes encoding for ribosomal protein in differentiated human tissues appears to be less concertedly regulated than previously reported (Bortoluzzi et al. 2001). Recent progress in ribosome research provides growing evidence that ribosomal proteins can also have a function during various cellular processes such as replication, transcription, RNA processing, DNA repair, and even inflammation; all these functions are independent of their own involvement in the protein biosynthesis (Wool 1996; Yamamoto 2000). In the present work, up-regulation of transcripts for several ribosomal proteins such as RPL13a, RPL19, RPL30, RPLP1, RPS24, and RPS26 has been observed. This latter has been described as a marker to differentiate either ozone or ultraviolet B radiation environmental stresses in plants (Brosché and Strid 1999). Whereas RPS4, RPL19, and RPS18 have been involved in regulation of the development (Wool 1996), RPL13A, RPS18, and RPS24 have been associated in the maturation of mucosal epithelia (Kasai et al. 2003). Moreover, the latter was markedly decreased in colorectal cancer (Kasai et al. 2003). Taken together, these observations may suggest that UN induce a perturbation in protein synthesis and offer a new putative way of investigation on cellular proliferation study after chronic UN exposure.

### Others Genes of Interest

ODC, described as the rate-limiting enzyme of polyamine biosynthesis and a marker of G_1_ phase, is down-regulated in long-term UN exposure. Recently, Fleck et al. (2003) also observed a decrease in *Odc* expression levels 10 weeks after a single injection of UN. Kramer et al. (2001) have showed that a depletion of polyamine pool, through inhibition of ODC, causes p21-mediated G_1_ cell cycle arrest, followed by development of a senescence-like phenotype and loss of cellular proliferative capacity. Thus, the decrease in *Odc* mRNA levels might be related to an arrest of the cell cycle after UN treatment. However, these data are inconsistent with the observed increase in protein biosynthesis–related genes. It has been previously reported that mammalian ODC protein has a very short half-life; its control is under negative feedback regulation by the polyamines, and its degradation is dependent on 26S proteasome complex (Hascilowicz et al. 2002). Interestingly, we noted an increase in proteasome subunit (*Psma7*) mRNA expression levels. Nevertheless, further study with added dimensions of time and doses may clarify the observed modest *Odc* mRNA expression levels for the group 2 and allow a best evaluation of uranium chronic exposure impact on its expression. Of particular interest, *Umod* (Tamm-Horsfall protein) was decreased in the present study. This protein is one of the most abundant in the renal tubule (Bachmann et al 1990). Moreover, expression levels of UMOD have been previously reported to decrease in ischemic-induced ARF (Yoshida et al. 2002). Unexpectedly, in previous work performed in our laboratory, we showed that its expression level was increased in UN-induced ARF. In addition, an up-regulation of *Umod* has been observed in the progression of nephrolithiasis (Katsuma et al. 2002). However, the role of this protein remains unclear and requires additional investigation. Finally, and perhaps more interestingly, TCTP, a cytoplasmic protein usually found in both normal and tumor cell lines, is overexpressed after UN long-term ingestion. It was identified as an antiapoptotic protein (Li et al. 2001). TCTP is associated with components of the translational machinery, the elongation factors implicated in tumor formation (Cans et al. 2003). Interestingly, we observed dose-dependent increases in *Tctp* mRNA levels using RT-PCR analysis. Further investigations are necessary to evaluate the implication of this protein in potentially adverse health effects.

**Figure 3:**
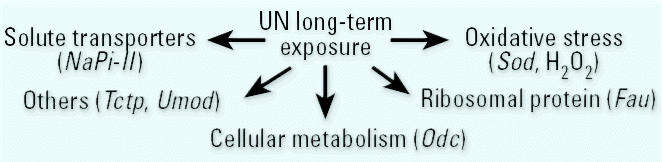
Cellular pathways triggered in response to UN long-term exposure. Some genes or molecules, which present an altered expression level after uranium ingestion, emphasize the implication of these cellular processes in UN long-term exposure (in parentheses).

In summary, by using SAGE, we elegantly demonstrated that UN chronic exposure induces changes in expression profiles. The present report provides the first evidence that UN alters the expression of numerous genes including those encoding for oxidative-stress–related proteins, ribosomal proteins, solute transporters, and genes involved in cellular metabolism or signal transduction (Figure 3). Although these molecular changes, resulting from a subclinical toxicity, do not systematically lead to kidney failure or overt illness, our results might constitute a determining step in the identification of sensitive biomarkers to prevent the development of a UN-induced renal injury. Moreover, although studying human biology is ideal, such studies are neither feasible nor ethical. Thus, the vast majority of current biomedical research is conducted using mice and rats. However, we must keep in mind that extrapolation to humans might have some bias because humans can be exposed to many compounds simultaneously, often on a chronic or intermittent basis. Thus, the use of throughput genomic approaches after long-term exposure to mixtures of toxicants might help in the assessment of interactions such as additivity, synergism, or antagonism. The comparison of gene expression profiles could help to identify putative new sensitive biomarkers of chronic nephrotoxicity and then evaluate the impact of environmental toxic contaminants on human health.

## Figures and Tables

**Table 1:** SYBR Green and TaqMan primer sequences used for RT-PCR reactions.

Gene symbol*^a^*	Gene name*^a^*	Accession no.*^a^*	Primer 5′→3′ sequence or assay ID	Amplicon size (bp)
Primers using SYBR Green detection				
*Hprt*	hypoxanthine phosphoribosyl transferase	NM_013556	Forward 5′-TTGCTGACCTGCTGGATTAC-3′ *^b^*	112
			Reverse 5′-CCCGTTGACTGATCATTACA-3′	
*Sod1*	superoxide dismutase 1	XM_128337	Forward 5′-TGGTGGTCCATGAGAAACAA-3′	75
			Reverse 5′-TCCCAGCATTTCCAGTCTTT-3′	
*Odc*	ornithine decarboxylase, structural	NM_013614	Forward 5′-TTGCCACTGATGATTCCAAA-3′	129
			Reverse 5′-CATGGAAGCTCACACCAATG-3′	
*Fau*	Finkel-Biskis-Reilly murine sarcoma virus	NM_007990	Forward 5′-GCTGGGAGGTAAAGTTCACG-3′	125
			Reverse 5′-TGTACTGCATTCGCCTCTTG-3′	
*Tctp*	translationally regulated transcript	NM_009429	Forward 5′-CCGGGAGATCGCGGAC-3′	92
			Reverse 5′-TTCCACCGATGAGCGAGTC-3′	
Primers using TaqMan technology				
*Hprt*	hypoxanthine phosphoribosyl transferase	NM_013556	Mm00446968m1*^c^*	
*Kap*	kidney androgen regulated protein	NM_010594	Mm00495104m1	
*NaPi-II*	solute carrier family 34, member 1	NM_011392	Mm00441450m1	
*Umod*	uromodulin	NM_009470	Mm00447649m1	

a From Applied Biosystems (http://myscience.appliedbiosystems.com/cdsEntry/Form/gene_expression_keyword.jsp).

b Primer 5′→3′ sequence.

c Assay ID – Applied Biosystems.

**Table 2:** Physiologic parameters in serum and urine and uranium amount in control group 0 and contaminated groups 1 and 2 after 4 months of daily contamination (mean ± SE).

	Group		
Parameter	0	1	2
Exposure (mg UN/L)	0	80	160
Kidney			
Weight (g)	0.47 ± 0.01	0.46 ± 0.01	0.47 ± 0.02
Uranium amount (μg/g)	0.16 ± 0.04	0.35 ± 0.02*	1.05 ± 0.21*
Serum			
Urea (mg/dL)	59 ± 5	57 ± 5	54 ± 7
Creatinine (mg/dL)	0.12 ± 0.02	0.23 ± 0.02*	0.25 ± 0.02*
Urine			
Glucose (g/L)	0.08 ± 0.03	0.08 ± 0.03	0.04 ± 0.01
γ -GT (U/L)	86 ± 44	94 ± 42	119 ± 66

*p < 0.05 versus control; *n* = 4.

**Table 3:** List of tags with significant variations in expression level induced by UN long-term ingestion (*p* < 0.05), their frequency, and their relevant accession number.

Tag sequence	Count		Gene name*^a^*	Accession no.*^a^*	Regulation*^b^*	Gene symbol*^a^*
	UN(−)	UN(+)				
**Apoptosis**						
GCTGCCAGGG	11	4	Bcl2-associated athanogene 1	NM_009736	−	*Bag1*
GAAAGCAATG	0	6	nerve growth factor receptor (TNFRSF16) associated protein 1	NM_009750	+	*Ngfrap1*
TGCCTTACTT	3	8	programmed cell death 6	NM_011051	+	*Pdcd6*
**Amino acid metabolism**						
CGTATCTGTA	4	10	D-amino acid oxidase	NM_010018	+	*Dao1*
CAGTTACAAA	1	6	glutamate dehydrogenase	NM_008133	+	*Glud*
TTTTACCTGC	0	8	glycine amidinotransferase (L-arginine:glycine amidinotransferase)	NM_025961	+	*Gatn*
CTACCACTGC	4	12	fumarylacetoacetate hydrolase	NM_010176	+	*Fah*
ATACTAACGT	40	24	ornithine decarboxylase, structural	NM_013614	−	*Odc*
AACAGAAAGT	1	8	phenylalanine hydroxylase	NM_008777	+	*Pah*
**Carbohydrate metabolism**						
GCAAACAAGA	11	18	isocitrate dehydrogenase 2 (NADP+), mitochondrial	NM_173011	+	*Idh2*
GTGCCATATT	12	26	isocitrate dehydrogenase 2 (NADP+), mitochondrial	NM_173011	+	*Idh2*
/CCAAATAAAA	17	31	lactate dehydrogenase 1, A chain	NM_010699	+	*Ldh1*
TGATATGAGC	33	12	lactate dehydrogenase 2, B chain	NM_008492	−	*Ldh2*
TTGTTAGTGC	70	89	malate dehydrogenase, soluble	NM_008492	+	*Mor2*
GCAATCTGAT	17	31	phosphoglycerate kinase 1	NM_008828	+	*Pgk1*
GCCCAGACCT	25	41	sorbitol dehydrogenase 1	NM_146126	+	*Sdh1*
GCTTGTGACG	1	8	transaldolase 1	NM_011528	+	*Taldo1*
**Cell adhesion**						
CTCTGACTTA	3	8	basigin	NM_009768	+	*Bsg*
GAGACTAGCA	4	10	transmembrane 4 superfamily member 8	NM_019793	+	*Tm4sf8*
**Immunity and defense**						
*Immunity*						
GTTCAAGTGA	4	12	Ia-associated invariant chain	NM_010545	+	*Ii*
TATCCTGAAT	14	2	lymphocyte antigen 6 complex, locus A	NM_010738	−	*Ly6a*
TTTTATGTTT	12	20	tumor necrosis factor, alpha-induced protein 1 (endothelial)	NM_009395	+	*Tnfaip1*
TATACATCCA	43	26	uromodulin	NM_009470	−	*Umod*
TGGGTTGTCT	151	174	translationally regulated transcript (21 kDa)	NM_009429	+	*Trt, Tpt1, Tctp*
*Antioxidant and free radical removal*						
CTATCCTCTC	297	341	glutathione peroxidase 3	NM_008161	+	*Gpx3*
CAGCTTCGAA	12	2	glutathione S-transferase, theta 2	NM_010361	−	*Gstt2*
AGAAACAAGA	7	18	superoxide dismutase 1, soluble	XM_128337	+	*Sod1*
TTGCTTCTAT	20	8	thioether S-methyltransferase	NM_009349	−	*Temt*
CATCAGCCTC	7	0	thioredoxin, mitochondrial	NM_019913	−	*Txn2*
**Lipid fatty acid and steroid metabolism**						
TCTCCTTAGC	0	10	ATP-binding cassette, subfamily D (ALD), member 3	NM_008991	+	*Abcd3*
TTAAGACCTG	9	18	crystallin, zeta	NM_009968	+	*CryZ*
TATAATAAAC	0	8	cytochrome P450, 2d9	NM_080006	+	*Cyp2d9*
TGTGTGGAAT	14	20	cytochrome P450, subfamily IV B, polypeptide 1	NM_007823	+	*Cyp4b1*
GGAGGGTGTG	4	10	phosphatidic acid phosphatase type 2c	NM_015817	+	*Ppap2c*
**Protein metabolism and modification**						
*Protein folding*						
CCTCCCTTTT	4	14	heat shock 10 kDa protein 1 (chaperonin 10)	NM_008303	+	*Hspe1*
*Protein biosynthesis*						
GATGTGGCTG	7	22	eukaryotic translation elongation factor 1 beta 2	NM_018796	+	*Esf1b2*
TCACCCAATA	36	49	eukaryotic translation elongation factor 2	NM_007907	+	*Eef2*
CTAATAAAGC	18	43	Finkel-Biskis-Reilly murine sarcoma virus (FBR-MuSV) ubiquitously expressed (fox derived)	NM_007990	+	*Fau*
TGTCATCTAG	7	14	laminin receptor 1 (67 kDa, ribosomal protein SA)	NM_011029	+	*Lamr1*
TGCTGGGATG	6	16	mitochondrial ribosomal protein S12	NM_011885	+	*Mrps12*
AGGTCGGGTG	7	14	ribosomal protein L13a		+	*Rpl13a*
TGGATCAGTC	47	66	ribosomal protein L19	NM_009078	+	*Rpl19*
CCAGAACAGA	7	20	ribosomal protein L30	NM_009078	+	*Rpl30*
GGCTTCGGTC	48	68	ribosomal protein, large, P1	NM_018853	+	*Rplp1*
GTGAAACTAA	36	45	ribosomal protein S4, X-linked	NM_009094	+	*Rps4x*
CTGGGCGTGT	3	8	ribosomal protein S15	NM_009091	+	*Rps15*
GTGGGCGTGT	0	6	ribosomal protein S15	NM_009091	+	*Rps15*
CAGAACCCAC	0	6	ribosomal protein S18	NM_138946	+	*Rps18*
GCCTTTATGA	4	10	ribosomal protein S24	NM_011297	+	*Rps24*
AACAGGTTCA	11	18	ribosomal protein S25	NM_024266	+	*Rps25*
TAAAGAGGCC	18	29	ribosomal protein S26	NM_013765	+	*Rps26*
*Proteolysis*						
GGTTAAATGT	1	8	cathepsin L	NM_009984	+	*Ctsl*
CAGCAAAAAA	33	41	kallikrein 5	NM_008456	+	*Klk5*
GAGAGTGTGA	6	14	kidney-derived aspartic protease-like protein	NM_008437	+	*Kdap*
CAGAATGGAA	14	29	peptidase 4	NM_008820	+	*Pep4*
AGGCGGGATC	3	8	proteasome (prosome, macropain) subunit, alpha type 7	NM_011969	+	*Psma7*
CAACAAACAT	3	10	protein C	NM_008934	+	*Proc*
GTAAGCAAAA	22	43	ubiquitin B	NM_011664	+	*Ubb*
**Signal transduction system, receptor**						
TGGGACTCAC	4	14	cholecystokinin A receptor	NM_009827	+	*Cckar*
AGAAAAAAAA	7	14	ciliary neurotrophic factor receptor	NM_016673	+	*Cntfr*
TGATTTTTGT	1	10	disabled homolog 2 (Drosophila)	NM_023118	+	*Dab2*
GGGCAAGCCA	4	14	estrogen-related receptor, alpha	NM_007953	+	*Esrra*
CATACGCATA	7	16	growth hormone receptor	NM_010284	+	*Ghr*
TTAAGAGGGA	12	0	transducer of ErbB-2.1	NM_009427	−	*Tob1*
**Transport**						
*Electron transport*						
GCTTTGAATG	20	35	ATPase inhibitor	NM_007512	+	*Atpi*
CCAGTCCTGG	12	24	ATP synthase, H+ transporting, mitochondrial F0 complex, subunit c (subunit 9), isoform 1	NM_007506	+	*Atp5g1*
GTTCTTTCGT	3	8	ATP synthase, H+ transporting, mitochondrial F0 complex, subunit c (subunit 9), isoform 2	NM_026468	+	*Atp5g2*
GCCGAGCATA	6	16	ATP synthase, H+ transporting, mitochondrial F0 complex, subunit f, isoform 2	NM_020582	+	*Atp5j2*
GATAGATAAT	3	8	ATP synthase, H+ transporting, mitochondrial F1 complex, alpha subunit, isoform 1	NM_007505	+	*Atp5a1*
CTAATAAAAG	33	45	cytochrome c oxidase, subunit IVa	NM_009941	+	*Cox4a*
TATTGGCTCT	53	74	cytochrome c oxidase, subunit VIIIa	NM_007750	+	*Cox8a*
AGGGCACTGG	3	8	cytochrome c oxidase, subunit XVII assembly protein homolog	AV158262	+	*Cox17*
CAGAATGTGC	3	8	NADH dehydrogenase (ubiquinone) 1 alpha subcomplex, 2	NM_010885	+	*Ndufa2*
TTATGAAATG	15	24	NADH dehydrogenase (ubiquinone) 1 alpha subcomplex, 1	NM_019443	+	*Ndufa1*
ACTGCTTTTC	1	10	NADH dehydrogenase (ubiquinone) 1 alpha subcomplex, 7	NM_023202	+	*Ndufa7*
*Ion transport*						
TTCTAGCATA	28	10	ATPase, Na+/K+ transporting, beta 1 polypeptide	NM_009721	−	*Atp1b1*
CTAGGTACTG	48	91	solute carrier family 34 (sodium phosphate), member 1	NM_011392	+	*Slc34a1*
ACAAATTATG	1	8	voltage-dependent anion channel 2	NM_011695	+	*Vdac2*
**Lipid fatty acid transport**						
GCTCTGATAC	0	8	sterol carrier protein 2, liver	NM_138508	+	*Scp2*
**Others**						
TGCTTTTACG	7	20	6-pyruvoyl-tetrahydropterin synthase/dimerization cofactor of hepatocyte nuclear factor 1 alpha (TCF1)	NM_025273	+	*Pcbd*
ATTACGGTGG	7	18	aldo-keto reductase family 1, member A4 (aldehyde reductase)	NM_021473	+	*Akr1a4*
AAGACCTATG	12	2	diazepam binding inhibitor	NM_007830	−	*Dbi*
CTCCTGCAGC	15	29	esterase 10	NM_016903	+	*Es10*
ATCTGACTCC	3	10	hemoglobin Y, beta-like embryonic chain	NM_008221	+	*Hbb*
TAAAGCAAAA	20	43	H2B histone family, member S	NM_023422	+	*Hist1h2bc*
GACTTCACGC	155	182	kidney androgen-regulated protein	NM_010594	+	*Kap*
GCACGAGCGT	7	0	low density lipoprotein receptor-related protein 2	XM_130363	−	*Lrp2*
TGCTGTGACC	9	16	membrane-associated protein 17 pending	NM_026018	+	*Map17-p*
TGTGCTTCCC	4	12	neural precursor cell expressed, developmentally down-regulated gene 8	NM_008683	+	*Nedd8*
TGAGCGCTGC	15	24	PDZ domain containing 1	NM_021517	+	*Pdzk1*
GGGGAGGGGG	7	0	pre B-cell leukemia transcription factor 2	NM_017463	−	*Pbx2*
GGCTGGGGGC	3	10	profilin 1	NM_011072	+	*Pfn1*
AAGTAAAGCG	6	12	SEC61, gamma subunit (S. cerevisiae)	NM_011343	+	*Sec61g*
CAGCCTGAGC	4	10	selenoprotein R	NM_013759	+	*Sepr*
TTTCCAGGTG	1	8	selenoprotein W, muscle 1	NM_009156	+	*Sepw1*

a From Applied Biosystems (http://myscience.appliedbiosystems.com/cdsEntry/Form/gene_expression_keyword.jsp.).

b +, up-regulation; −, down-regulation.
